# Emerging Preservation Techniques for Controlling Spoilage and Pathogenic Microorganisms in Fruit Juices 

**DOI:** 10.1155/2014/758942

**Published:** 2014-09-22

**Authors:** Kamal Rai Aneja, Romika Dhiman, Neeraj Kumar Aggarwal, Ashish Aneja

**Affiliations:** ^1^Vaidyanath Research, Training and Diagnostic Centre, Kurukshetra 136118, India; ^2^Department of Microbiology, Kurukshetra University, Kurukshetra 136119, India; ^3^University Health Centre, Kurukshetra University, Kurukshetra 136119, India

## Abstract

Fruit juices are important commodities in the global market providing vast possibilities for new value added products to meet consumer demand for convenience, nutrition, and health. Fruit juices are spoiled primarily due to proliferation of acid tolerant and osmophilic microflora. There is also risk of food borne microbial infections which is associated with the consumption of fruit juices. In order to reduce the incidence of outbreaks, fruit juices are preserved by various techniques. Thermal pasteurization is used commercially by fruit juice industries for the preservation of fruit juices but results in losses of essential nutrients and changes in physicochemical and organoleptic properties. Nonthermal pasteurization methods such as high hydrostatic pressure, pulsed electric field, and ultrasound and irradiations have also been employed in fruit juices to overcome the negative effects of thermal pasteurization. Some of these techniques have already been commercialized. Some are still in research or pilot scale. Apart from these emerging techniques, preservatives from natural sources have also shown considerable promise for use in some food products. In this review article, spoilage, pathogenic microflora, and food borne outbreaks associated with fruit juices of last two decades are given in one section. In other sections various prevention methods to control the growth of spoilage and pathogenic microflora to increase the shelf life of fruit juices are discussed.

## 1. Introduction

Consumer demand for nutritious foods such as fresh cut fruits and unpasteurized fruit juices has increased in the last decades owing to their low content of sodium, cholesterol and fat and high concentration of vitamin C, polyphenols, and antioxidants that play important role in the prevention of heart diseases, cancer, and diabetes [1–4]. Juice is defined as unfermented but fermentable juice, intended for direct consumption, obtained by the mechanical process from sound, ripe fruits, and preserved exclusively by physical means. The addition of sugars or acids can be permitted but must be endorsed in the individual standard [5–7] Increased consumption of fruit juices has direct influence on economy in positive way but in negative way also when food borne disease outbreaks and spoilage problems occur [8]. Juices have become frequent vehicle for transmitting pathogens such as enterohaemorrhagic* Escherichia coli* O157,* Salmonella*, and* Cryptosporidium* [9]. Several emerging spoilage microorganisms also have great concern in fruit juice industry; for example,* Alicyclobacillus acidoterrestris* has been isolated from several juices and juice products with reported occurrence between 14.7% and 18.3%.* Propionibacterium cyclohexanicum* and heat resistant species of mycelial fungi such as* Byssochlamys fulva*,* B. nivea*, and* Neosartorya fischeri* and species of* Talaromyces* have also been reported to spoil fruit juices [10–13]. There is tremendous increase in the food borne disease outbreaks associated with the consumption of fruit juices [14].

Keeping in view the threat challenge posed by spoilage and pathogenic microorganisms to both the fruit juice industry and public health authorities, several guidelines have been published by national food standard agencies, such as HACCP and FDA, to control or reduce the incidence of food borne disease outbreaks or spoilage [8]. For prevention of these microorganisms in fruit juices, thermal treatment is effective method for microbial inactivation but it may produce some undesirable effects on foods such as loss of nutrients and reduction of fresh like flavor [15, 16]. New technologies such as high hydrostatic pressure (HHP), high pressure homogenization (HPH), pulsed electric field (PEF), ultrasound, and irradiations have been developed to maintain nutritional and sensory quality of fruit juices [17, 18].

Chemical preservatives, such as sodium benzoate and potassium sorbate, are commonly used in fruit juices and beverages to extend their shelf life [11]. However, consumer demand for fresh and safe foods without chemically synthesized preservatives leads to increase the interest in use of food preservatives from natural sources [19]. Natural preservatives such as bacteriocins, organic acids, essential oils, and phenolic compounds have been used in some food products [19, 20].

## 2. Microorganisms Involved in Spoilage

Change in the appearance, smell or taste of a food that makes it unacceptable to the consumer is called food spoilage [21]. Spoilage of fruit and vegetable juices is primarily owing to the proliferation of their natural acid tolerant and osmophilic microflora [22]. Fresh fruit juices are more susceptible to spoilage because fluid contents are in touch with air and microorganisms from the environment during the time of handling [4]. Yeasts, heat sensitive moulds, and lactic acid bacteria are indicator for the quality of raw materials. Heat resistant fungi and other spore forming bacteria such as* Clostridium pasteurianum* and* Bacillus coagulans* are used as targets for fruit juice pasteurization processes [8].

## 3. Yeasts

Yeasts have the ability to grow at low pH, high sugar concentration, and low water activity. Fruit juices are generally rich in simple carbohydrates and complex nitrogen sources and hence are ideal substrates for yeasts [22]. Over 110 species of yeasts have been reported to be associated with food and food products [23] and are dominating contaminants in fruit juices ranging from 1.0 to 6.83 log 10 cfu/mL [24]. The presence of yeasts in fruit juices may result from failures in fruit juice pasteurization and failure in sanitation practices [8]. Spoilage by yeasts in fruit juices is characterized by formation of CO_2_ and alcohol. Yeasts may also produce turbidity, flocculation, pellicles, and clumping. Yeasts also produced pectinesterases which degrade pectin causing spoilage, organic acids, and acetaldehyde, which contribute for a “fermented flavor,” may also be formed [6, 25].

*Pichia*,* Candida*,* Saccharomyces*, and* Rhodotorula* are the genera mainly responsible for the spoilage of fruit juices; species frequently isolated are* Pichia membranifaciens*,* Candida maltosa*,* C. sake*,* Saccharomyces bailii*,* S. bisporus*,* S. cerevisiae*,* S. rouxii*,* S. bayanus*,* Brettanomyces intermedius*,* Schizosaccharomyces pombe*,* Torulopsis holmii*,* Hanseniaspora guilliermondii*,* Schwanniomyces occidentalis*,* Dekkera bruxellensis*,* D. naardenensis Torulaspora delbrueckii*, and* Zygosaccharomyces microellipsoides* [7]. Major yeast species found in citrus juices are* Candida parapsilosis*,* C. stellata*,* Saccharomyces cerevisiae*,* Torulaspora delbrueckii*, and* Zygosaccharomyces rouxii* [26]. Some of these species are sensitive to thermal pasteurization treatment applied to fruit juices [6].

### 3.1. Yeasts Resistant to Preservatives

Resistance to preservatives is a great threat to the stability of fruit juices [27]. Examples of yeasts resistant to preservatives include* Zygosaccharomyces bailli*,* Candida krusei*,* Saccharomyces bisporus*,* Schizosaccharomyces pombe*, and* Pichia membranifaciens* [27, 28]. Resistance to preservatives has been attributed to the ability of cells to tolerate chronic intracellular pH drops by phosphofructokinase enzyme [6].* P. membranifaciens* is resistant to heat, moderate amount of salt, SO_2_, sorbic, benzoic and acetic acid; hence, it is considered as target microorganism for optimization of thermal pasteurization [7].

## 4. Moulds

Moulds are aerobic which grow at low pH and high sugar concentration. In response to thermal treatment, moulds are divided into two categories: heat labile and heat resistant [29, 30]. Former kinds produce mycelial mats in juice and adhere to the package interior, carton seams and produce musty and stale off-flavours. Juice cloud loss occurs through the activity of pectinesterases [6, 29]. The dominant moulds recorded in fruit juices belong to* Penicillium* sp.,* Cladosporium* sp.,* Aspergillus niger*,* A*.* fumigatus*,* Botrytis* sp., and* Aureobasidium pullulans* [25].* Rhizopus *and* Mucor* are also associated with spoilage of fresh fruits and vegetables [30]. Among these, some moulds produce mycotoxins which are of great threat to human health. Major mycotoxins associated with fruit juices are byssochlamic acid (*Byssochlamys fulva*,* B. nivea*), patulin (*B. fulva*,* B. nivea*, and* P. expansum*), ochratoxin (*Aspergillus carbonarius*), and citrinin (*Penicillium expansum*,* P. citrinum*) [29, 31]. Presence of patulin in fruit juices is indicator of poor quality of fruits used in processing of juices [32].

### 4.1. Heat Resistant Moulds

The moulds, which able to survive at 85°C for 4.5 minutes, low oxygen tension, low pH (3.0–4.5) and produce pectinolytic enzymes, have an influence on juice stability [24]. Some notable species are* Byssochlamys fulva*,* B. nivea*,* Neosartorya fischeri*, and* Talaromyces *[6, 24, 33]. These moulds survive commercial heat pasteurization treatment, usually applied to fruits and fruit products because of the presence of heat resistant ascospores [6, 25, 34]. Heat resistance also depends upon the fruit product. As the concentration of sugar increases, heat resistance in microorganisms also increases [6, 24, 33].

The presence of heat resistant fungi such as* Paecilomyces variotii*,* Aspergillus tamari*,* A. flavus*, and* A. ochraceus* has been reported in sixty packaged Nigerian fruit juices consisting of mango, pineapple, orange, and tomato [35]. Chlamydospores, sclerotia, and aleurospores are the resistant structures/spores produced by these moulds [34, 36].

A pasteurization temperature for fruit and fruit products often tested is 90°C for 3 minutes. This treatment may not be adequate to inactivate ascospores of* Byssochlamys fulva*,* Neosartorya fischeri*, and* Talaromyces* species [24]. Salomão et al. [34] reported that the thermal tolerance level of the ascospores of heat resistant moulds varies from strain to strain and with the composition of the heating medium as explained in Table 1.

The sources of contamination of these ascospores of heat resistant fungi found in fruit juices are soil, especially in case of grapes, passion fruits, pineapples, mangoes, strawberries, and other berries [6]. Other sources of contamination are processing facilities, air, utensils, fields, and orchards [24].

## 5. Bacteria

Bacteria are present in low numbers on fresh fruits and vegetables due to low pH. Acid tolerant bacteria such as heterofermentative lactic acid bacteria, acetic acid bacteria,* Erwinia* sp.,* Enterobacter* sp.,* Clostridium*,* Alicyclobacillus acidoterrestris*,* Propionibacterium cyclohexanicum*,* Pseudomonas* sp., and* Bacillus *sp. have been reported as deteriorative in fruit juices [7, 19] which are discussed here.

### 5.1. Lactic Acid Bacteria (LAB)

Lactic acid bacteria are gram positive, rod shaped, and catalase negative. Heterofermentative lactic acid bacteria were reported as the most important group of spoilage microorganisms in fruit juices [25].* Lactobacillus* and* Leuconostoc* are the two taxa frequently isolated from fruits and spoiled fruit juices [6]. They produce lactic acids in fruit juices along with lesser amount of acetic and gluconic acids, ethanol and CO_2_, but some species of lactic acid bacteria such as* L. mesenteroides* ssp.* cremoris*,* Leuconostoc paramesenteroides*, and* Leuconostoc dextranicum* are more prominent as they produce diacetyl and acetoin as metabolites in spoiled fruit juices, contributing to buttery or butter milk off flavor to citrus juices [6, 13, 25].

### 5.2. Acetic Acid Bacteria

Acetic acid bacteria belong to three taxa, namely,* Acetobacter*,* Gluconobacter*, and* Gluconacetobacter*. These are gram negative or gram variable, aerobic, ellipsoidal to rod shaped cells that can occur in chains, single or in pairs and are among the main spoilage bacteria because they have the ability to grow at relatively low pH and low nutrient levels. Production of sour and vinegar like flavours in fruit juices is due to the formation of acetic acid by these bacteria [6, 25, 37].

### 5.3. Alicyclobacilli

In recent years,* Alicyclobacillus* a thermoacidophilic, endospore producing bacterium has emerged as major concern to the beverage industry worldwide as many high concentrated fruit products which are valuable semiprepared food components to the bakery, dairy, canning, baby foods, and distilling and beverage industries have been found to be contaminated with these spoilage microbes [13]. The thermoacidophilic nature and presence of highly resistant endospores is responsible for their survival during the production of concentrated fruit products. Soil is considered to be the main source of contamination of fresh fruits during harvesting. Some alicyclobacilli are soil borne microbes [38–42]. This bacterium was first classified as* Bacillus acidocaldarius* followed by* B. acidoterrestris* and now assigned to new genus* Alicyclobacillus* [6, 10, 12, 13, 33, 43, 44].

Of the over 20 species of* Alicyclobacillus* isolated from different environments. Some of them are* Alicyclobacillus acidocaldarius*,* A. hesperidium*,* A. acidophilus*,* A. cyclohaptanicus*,* A. fastidious*, and* A. pomorum* have been implicated in spoilage incidents in high acid fruit and vegetable products [45].* Alicyclobacillus acidoterrestris* has emerged as new spoilage bacterium for commercialized fruit juices that can survive pasteurization at 95°C for 2 minutes and can spoil heat treated fruit juices by the formation of taint chemicals (guaiacol and halophenolic) [13, 46].

Pathogenicity of* Alicyclobacillus acidoterrestris* and* A. caldarious* strains have extensively been studied [38].* Alicyclobacillus* contains *ω*-alicyclic fatty acids (*ω*-cyclohexane and *ω*-cycloheptane fatty acids) in their cell membrane that are responsible for heat resistance of* Alicyclobacillus* by forming a protective coating with strong hydrophobic bonds. These hydrophobic bonds stabilize reduced membrane permeability in extreme and high temperature environments [43, 44]. Another factor contributing to the heat stability of* Alicyclobacillus* is its endospores along with presence of heat stable proteins and mineralization by divalent cations especially calcium-dipicolinate complex [44, 47, 48].

Contamination of* Alicyclobacillus* in fruit juices results from sources like soil, water, and processing facilities [44]. Spoilage of fruit juices by* Alicyclobacillus* is difficult to detect because it does not produce any visible changes such as gas during growth and incipient swelling of containers does not occur so that spoilage in retail products cannot be noticed [33, 49]. It produces a smoky, medicinal, and antiseptic off-odour associated with guaiacol. Other compounds such as 2,6-dibromophenol and 2,6-dichlorophenol have also been found [46, 50, 51]. Endospores of* Alicyclobacillus* have D values in the range of 16–23 minutes at 90°C, greater than the pasteurization treatments applied in fruit juice processing [12]. Hence, Silva and Gibbs [33] suggested that* Alicyclobacillus *is to be designated as the target microbe in the design of pasteurization processes for acidic foods and beverages.

#### 5.3.1. *Propionibacterium cyclohexanicum*

*Propionibacterium cyclohexanicum*, a gram positive, acid tolerant, heat resistant, nonmotile pleomorphic rod shaped bacterium first isolated from spoiled orange juice in 1993 [52]. It possesses *ω*-cyclohexyl undecanoic acid in cell membrane as* Alicyclobacillus* genus but lacks the production of endospores [11]. Walker and Phillips [10] reported that* Propionibacterium cyclohexanicum* survives at 95°C for 10 minutes in orange juice and hence would survive treatments commonly used in pasteurization process used in fruit juice industry.

#### 5.3.2. *Streptomyces*

*Streptomyces* is a gram positive, filamentous rod shaped soil bacterium, possesses spores in chains.* Streptomyces griseus* is frequently found in spoiled apple juice. This bacterium enters into fruit juices through poorly washed fruits that are contaminated with soil [53]. It produces earthy like off-flavour in juices attributable to the presence of compounds like geosmin, 2-methyl isoborneol, and 2-isobutyl-3-methoxy pyrazine [8].

#### 5.3.3. *Bacillus*

*Bacillus coagulans*,* B. marcesens*, and* B. polymyxa* are gram positive, rod shaped, and endospore producers often spoil various fruit juices [54].* Bacillus coagulans* spoils canned tomato juice and vegetable products. It causes flat sour spoilage in juice [6, 13, 33, 55].

#### 5.3.4. *Clostridium*

Two species of* Clostridium* mainly* C. pasteurianum* and* C. butyricum*, gram positive, anaerobic endospore forming bacterium, have been isolated at low pH of fruit juices [54].

## 6. Pathogenic Microorganisms

Fruit surfaces can be contaminated with feces and feces present on fruit surfaces can contaminate the washing water and permit the internalization of food borne pathogens which help in their survival under the acidic conditions of fruit juices [8, 56]. Some strains of* Escherichia coli*,* Shigella*, and* Salmonella* may survive for several days and even weeks in acidic environment by regulating their internal pH that maintained at neutral pH by combination of passive and active mechanisms [9].

*Shigella flexneri* and* S. sonnei* survive in apple juice (pH 3.3) and tomato juice (pH 4.0) at 7°C for at least 14 days [57]. Sospedra et al. [14] reported the presence of* Salmonella* sp. and* Staphylococcus aureus* in orange juice extracted by squeezing machine used in restaurants. Several studies carried out by researchers on pathogenic microorganisms associated with street vended unpasteurized fruit juices [58–62] as summarized in Table 2.

Because of the presence of pathogens in fruit juices, the food borne outbreaks associated with consumption of fruit juices have been increased [9, 14, 19, 63, 64]. Fruit juice borne outbreaks of last two decades from 1991–2010 are summarized in Table 3. Several outbreaks associated with consumption of fruit juices have been reported maximum in year 1999 (5) [59, 65–67] and 1996 (4) [68–70].* E. coli* O157:H7 is associated with large number of outbreaks attributable to consumption of unpasteurized apple juice.* Salmonella* is main causal organism for outbreaks related to unpasteurized orange juice.* Clostridium botulinum* is reported from homemade as well as pasteurized carrot juice.* Vibrio cholorae* has been reported for outbreak in India by the consumption of unpasteurized sugarcane juice (Table 3). In year 1999, 423 people in the USA and Canada and 405 people in Australia were affected by consuming unpasteurized orange juice [59].

## 7. Isolation of Spoilage Microorganisms from Fruit Juices

Microbiological examination of fruit juices is done by serial dilution pour plate methods [71]. For total aciduric microbial populations, orange serum agar (pH—5.5, Orange Serum 200 mL/L; Yeast Extract—3 g/L; Enzymatic Digest of Casein—10 g/L; Dextrose—4 g/L; Potassium Phosphate—2.5 g/L; Agar—17 g/L) is used. Potato dextrose agar (PDA) (pH—3.5, potato—200 g/L; dextrose—20 g/L, agar—15 g/L), PDA with antibiotics (pH—5.6), malt extract agar (MEA) (pH—5.5; Malt extract—30 g/L; Mycological peptone—5 g/L; Agar 15 g/L) supplemented with chloramphenicol (100 mg/L, added before autoclaving) are used for enumeration of yeasts and moulds at incubation temperature of 25°C for 3 and 7 days for yeasts and moulds, respectively [25].

Isolation of yeasts resistant to preservatives from fruit juices is done by plating on MEA with 0.5% acetic acid and tryptone glucose yeast extract agar (TGY) (pH—7.0, Casein enzymatic hydrolysate—5 g/L; Yeast extract—3 g/L; Glucose—1 g/L; Agar—15 g/L) with 0.5% acetic acid at 25–30°C for 3–5 days. For the isolation of heat resistant fungi, fruit sample must be treated in water bath at 75°C to 80°C for 1.5 hour and isolated by using PDA, OSA, and MEA media [25].

PDA at pH 3.7 and OSA at pH 5.5 at an incubation temperature of 50°C for 3 days are the best isolation media for the detection and isolation of* Alicyclobacillus* sp. from fruit juices and concentrates [46]. Further identification of moulds and yeasts is done by using methods and media described in Samson et al. [72] and identification of bacterial isolates is done by using methods described in* Bergey's Manual of Systematic Bacteriology* [73] as well as by using advanced DNA-sequencing techniques.

## 8. Prevention of Spoilage and Pathogenic Microorganisms in Fruit Juices

There are various techniques to prevent pathogenic as well as nonpathogenic microflora such as chilling, freezing, water activity, modified atmosphere packaging, pasteurization, nonthermal physical techniques, and by addition of natural antimicrobials [19]. The most common method to inactivate microorganisms and enzymes for increasing the shelf life of fruit juices is by thermal processing; however, loss of original taste and flavor compounds occur in fruit juices. These negative effects have motivated a great interest in the development of new technology that offer advantages of using low processing temperatures, low energy consumption, and high retention of nutritional and sensory properties of the food and improving its microbiological quality [17].

## 9. Thermal Pasteurization

FDA has recommended 5 log_10_ *⁡* reduction of infection pathogens in a fruit juice which can be achieved by pasteurization at 90–95°C for 4–10 seconds [4, 6]. This pasteurization temperature is effective against* E. coli* and* Salmonella* [50] but is not effective against ascospores of heat resistant fungi [34, 74] and heat resistant bacteria [13, 44, 49, 50]. In addition, the thermal pasteurization damages nutritional and physiochemical properties of fruit juices [4].

Nonthermal preservative methods are receiving good attention because of their potential for quality and safety improvement of food [15]. Some of the nonthermal processes used in food industries are high intensity pulsed electric field (HIPEF), high hydrostatic pressure (HHP), high pressure homogenization (HPH), ultraviolet (UV), ultrasound, and irradiation. These novel nonthermal technologies have the ability to inactivate microorganisms at ambient or near ambient temperatures, thus avoiding the deleterious effect of heat has on flavor, colour, and nutrient value of foods mean effect of thermal treatment on fruit juices [84]. Apart from these methods, sodium benzoate and potassium sorbate are two of the most commonly used chemical preservatives to increase the shelf life of fruit juices. But consumer demand for safe, natural, and environmental friendly food preservatives has been increasing. Natural antimicrobials such as bacteriocins, lactoperoxidase, herbs, leaves, oils, and spices have shown potential for use in some food products [18]. Some of the common nonthermal methods used in fruit juice industry are described below.

## 10. High Hydrostatic Pressure (HHP)

High hydrostatic pressure is commercially used worldwide for a variety of foods such as cooked meat, shellfish, fruit, and vegetable juices, sauces, and dips [75]. In this process, fruit juices are subjected to 400 MPa pressure for a few minutes at 20°C or below which is sufficient to reduce the numbers of spoilage microorganisms such as yeasts, moulds, and lactic acid bacteria [75, 76]. The HHP treatment has a lethal effect on microorganisms by affecting their cell membrane along with inactivation of some key enzymes which are involved in DNA replication and transcription processes [15, 75]. Bacterial spores are resistant to HHP treatment and they can survive up to pressure of 1000 MPa [76]. This process has great potential to reduce the microbial load of fruit juices and increase the shelf life of fruit juices (Table 4).

## 11. High Pressure Homogenization (HPH)

It involves the pumping of liquid through homogenizing valve at high pressure over 100 MPa. It produces high turbulence and shear along with compression, acceleration, and pressure drop result in the breakdown of particles and dispersion throughout the product. After homogenization, the particles of uniform size in the range from 0.2 *μ*m to 2 *μ*m are obtained [16]. In the past, HPH was purposed as a suitable method for the stabilization of dairy products but in last decades it has been suggested for its use for prolongation of the shelf life of fruit juices [22]. HPH inactivates microorganisms by damaging their structural integrity coupled with sudden rise in temperature produced in this process [75].  Table 5 summarizes the effect of HPH on the reduction of microbial load of fruit juices.

## 12. Pulsed Electric Field (PEF)

PEF involves the application of short duration and high intensity electric field pulses. The fluid foods are placed between two electrodes in batch and continuous flow treatment [17]. This process inactivates microorganisms and enzymes with only small increase in temperature [77], affects the cell membrane of microorganisms by electroporation which leads to leakage of cytoplasmic content from cells [78, 79]. The work carried out on the inactivation of microorganisms in fruit juices by PEF treatment is summarized in Table 6.

## 13. Ultraviolet Technology (UV)

Ultraviolet technology has been utilized in the food industry to disinfect water and effectively destroy microorganisms on surfaces and packaging [80]. Ultraviolet radiation involves the use of radiation from electromagnetic spectrum from 100 to 400 nm. It is classified as UV-A (320–400 nm), UV-B (280–320 nm), and UV-C (200–280 nm) [81]. UV-C is effective against bacteria and viruses [81]. UV treatment is performed at low temperature. 254 nm wavelength of UV light is widely used in juice and beverage industry [18]. UV-C light inactivates microorganisms by damaging their DNA that absorbs UV light from 200 to 310 nm. UV creates the pyrimidine dimers which prevent microorganisms from replicating and thus rendering them inactive [81]. Novel works carried out on the effect of UV-C on the microbial load in different fruit juices have been given in Table 7.

## 14. Ultrasound

Power ultrasound has been identified as potential technology to meet US Food and Drug Administration (USFDA) requirement of 5 log reduction of* Escherichia coli* in fruit juices. High power ultrasound causes bubble cavitation in a liquid due to pressure changes. These resultant microbubbles collapse violently in the succeeding compression cycles of propagated ultrasonic waves resulting in localized high temperature up to 5000 K, pressures up to 50,000 kPa, and high shearing effects causing breakdown of cell walls, disruption of cell membranes, and damage of DNA of microorganisms [18, 82, 83]. Destruction of microorganisms in fruit juices by ultrasound which has been studied by various researchers is summarized in Table 8.

Nonthermal processes are to be used with caution in juices because they are not without harmful effects such as high hydrostatic pressure method which can alter the structure of protein and polysaccharide causing changes in the texture, physical appearance, and functionality of foods. High intensity ultrasound can denature proteins and produce free radicals that can adversely have an effect on the flavor of fruit based or high fat foods. Ultraviolet treatment is difficult to apply on fruit juices because of low UV transmittance through the juice because of high suspended and soluble solids [81]. Emerging nonthermal technology also has been so energy expensive or costly to be practical for use in food processing [84].

## 15. Preservatives

Another method to prevent microbial contamination in juice is by use of preservatives. Some chemical preservatives such as sodium benzoate and potassium sorbate are often used to prevent microbial spoilage of fruit juices [8, 18]. Consumers relate synthetic preservatives as artificial products resulting in rejection of this type of food processed [8] so demands for preservatives which have natural origin have increased drastically.

Most of these natural antimicrobials have been considered as generally recognized as safe (GRAS). Natural antimicrobials are obtained from three natural sources such as plant (herb, spices, essential oil, and vanillin), animal (lactoperoxidase, lysozyme, chitosan), and microbes (bacteriocins) [19]. The antimicrobial activity of these compounds has been studied in fruit juices by many researchers as detailed given in Table 9 and employ different mechanisms to inactivate microorganisms.

Lactoperoxidase system produces hypothiocyanite (OSCN^−^) and hypothiocyanous acid (HOSCN), that possess antimicrobial effect by the oxidation of thiol group (^−^SH) of cytoplasmic enzymes, damage to outer membrane, transport systems, glycolytic enzymes, and nucleic acids [85]. But lysozyme attack the bacterial peptidoglycan cell wall and are more effective against gram positive than gram negative bacteria because former contain about 90% peptidoglycan and later contain 5% to 10% [19].

Chitosan is effective against microorganisms due to its positive charge amino group at C–2 which can create polycationic structure and interact with anionic components such as lipopolysaccharide and proteins of cell surface; this binding disrupts the integrity of the outer membrane resulting in leakage of intracellular components [18]. Many herbs and plant extracts have broad spectrum activity against microorganisms [4, 86]. Essential oils are a group of terpenoids, sesquiterpenes, and possibly diterpenes with different groups of aliphatic hydrocarbons, acids, aldehydes, acyclic esters, or lactones. The antimicrobial activity of essential oil is not attributed to one specific mechanism, but there are several targets in the cell [87–89]. Hydrophobicity of essential oil enables them to partition in the lipids of bacterial cell membrane and mitochondria, disturbing the structures and rendering them more permeable [89].

Nisin possess narrow antimicrobial spectrum inhibiting only gram positive bacteria. It forms pore in cytoplasmic membrane of bacteria resulting in depletion of proton motive force and loss of cellular ions, amino acids, and ATP [19].

## 16. Synergistic Effect of Physical and Natural Antimicrobials

Food antimicrobials are generally biostatic and are not biocidal. Hence their effects on foods are limited. On the other hand use of combinations of antimicrobials [19] and antimicrobial along with nonthermal methods is effective against pathogenic and spoilage microorganisms. This combination improves the lethal effects of nonthermal processing and reduces the severity of nonthermal methods. So the combinations of these techniques could provide synergistic effects on prolonging the shelf life of fruit juices mentioned in Table 10 and potentially as best option for traditional pasteurization methods [8, 18].

## 17. Conclusion

The demand for fruit juices has been increasing due to their health benefits. Due to change in dietary, social habits and preservation methods have led to increase in disease outbreaks linked mainly to fresh fruit juices in recent years. Pasteurization of fruit juices is very effective against a pathogenic and several spoilage microorganisms, nonetheless, sensory and nutritional properties are affected. To meet the demands for nutritious and safe foods has resulted in increased interest in nonthermal preservation techniques. The nonthermal methods described in this review have the potential to meet 5 log microbial reductions. However, only high pressure processing has been used on pilot scale. There is also need for other nonthermal methods tested on pilot scale so they become alternative of pasteurization of fruit juices.

Application of different natural antimicrobials of animal, plant, and microbial origins directly or indirectly added to fruit juices effectively reduce or inhibit pathogenic and spoilage microorganisms. Thus they also represent good alternative of thermal processing of fruit juices.

In future, the combination of nonthermal methods and natural antimicrobial compounds would be new trend of preservation of fruit juices that improve the microbiological quality while having the lowest impacts on the organoleptic properties.

## Figures and Tables

**Table 1:** Thermal tolerance level of heat resistant moulds.

Heat resistant moulds	Thermal tolerance level	References
*Talaromyces flavus *	100°C for 5 to 12 minutes in many fruit syrups	[24]
*Byssochlamys fulva *	86° to 88°C for 30 minutes	[24]
*Paecilomyces variotii, Fusarium *sp.	95°C for 10–20 seconds	[90]

**Table 2:** Human pathogens isolated from street sold unpasteurized fruit juices.

Place	Fruit juice	Pathogens	Reference
Vishakhapatnam (India)	Orange, pomegranate, mango, pine apple, and grape	Faecal coliforms and faecal streptococci	[58]

Mumbai (India)	Sugarcane, lime, and carrot	*Vibrio cholerae, Escherichia coli, *and *Staphylococcus aureus *	[59]

Jimma town (South west Ethiopia)	Avocado, papaya, and pine apple	*Klebsiella, Enterobacter*, and *Serratia *	[60]

Nagpur (India)	Pine apple, sweet lime, and carrot juice	*Salmonella*, Coliforms, and * S. aureus *	[61]

Amravati (India)	Apple, orange, pineapple, pomegranate, sweet lemon, and mix fruit	*Salmonella, *Coliforms*, S. aureus, Pseudomonas*, and* Proteus *	[62]

**Table 3:** Fruit juice borne outbreaks caused by pathogenic bacteria from 1991 to 2010.

Type of fruit juices	Pathogens	Year	Country	Venue	Number of cases (deaths)	Reference
Unpasteurized apple juice	*Escherichia coli* O157:H7	1991	USA	Small cider mill	23 (0)	[91]

Unpasteurized orange juice	*Enterotoxigenic E. coli *	1992	India	Roadside vendor	6 (0)	[92]

Unpasteurized apple juice	*Cryptosporidium *	1993	USA	School	213 (0)	[93]

Carrot homemade juice	*Clostridium botulinum *	1993	USA	Home	1 (0)	[94]

Unpasteurized orange juice	*Salmonella gaminara, S*. *hartford,* and *S. rubislaw *	1995	USA	Retail	63 (0)	[95–97]

Unpasteurized orange juice	*Shigella flexneri *	1995	South Africa	Restaurant	14 (0)	[98]

Unpasteurized apple juice	*C. parvum *	1996	USA	Small cider mill	31 (0)	[68]

Unpasteurized apple juice	*E. coli* O157:H7	1996	USA	Small cider mill	14 (0)	[68]

Unpasteurized apple juice	*E. coli* O157:H7	1996	USA	Small cider mill	6 (0)	[70]

Unpasteurized apple juice	*E. coli* O157:H7	1996	Canada, USA	Retail	70 (1)	[69, 99]

Unpasteurized apple juice	*E. coli* O157:H7	1998	Canada	Farm/home	14 (0)	[100]

Unpasteurized apple juice	*E. coli *O157:H7	1999	USA	Not reported	25 (0)	[67]

Unpasteurized orange juice	*S*. *muenchen *	1999	Canada, USA	Restaurant	423 (1)	[65]

Unpasteurized orange juice	*S*. *anatum *	1999	USA	Roadside stand	6 (0)	[66]

Unpasteurized orange juice	*S*. *typhimurium *	1999	Australia	Retail	405 (0)	[59]

Unpasteurized sugar cane juice	*Vibrio cholerae *	1999	India	Not reported	—	[59]

Unpasteurized orange juice	*S*. *enteritidis *	2000	USA	Retail and food service	88 (0)	[101]

Unpasteurized apple juice	*C. parvum *	2003	USA	Farm/retail	144 (0)	[102]

Unpasteurized apple juice	*E. coli* O111 and *C. parvum *	2004	USA	Farm/home	212 (0)	[102]

Unpasteurized orange juice	*S. typhimurium* and* S. saintpaul *	2005	USA	Retail and food service	152 (0)	[103]

Unpasteurized sugar cane juice	*Trypanosoma cruzi *	2005	Brazil	Roadside kiosk	25 (3)	[104]

Pasteurized carrot juice	*C. botulinum *	2006	USA	Retail	4 (0)	[105]

Unpasteurized apple juice	*E. coli* O157:H7	2007	USA	Not reported	9 (0)	[67]

Unpasteurized apple juice	*E. coli* O157:H7	2008	USA	Retail	7	[67]

Unpasteurized orange juice	*S. panama *	2008	Netherlands	Not reported	33 (0)	[7]

Unpasteurized apple juice	*E. coli* O157:H7	2010	USA	Fair	7 (0)	[106]

**Table 4:** Effect of high hydrostatic pressure on microorganisms in fruit juices.

Fruit juice(s)	Target microorganisms	Treatment parameters	Log reduction	References
Orange juice	*Escherichia coli* O157:H7	550 MPa, 30°C, 5 min	6	[107]

Apple juice	*E. coli *29055	400 MPa, 25°C	>5	[108]

Apricot, sour cherry, and apple juices	*Staphylococcus aureus* *E. coli *O157:H7, *Salmonella enteritidis *	350 MPa, 30°C, 5 minutes	>5	[109]

Apple juice	*E. coli*, *Listeria innocua*, and *Salmonella *	545 MPa, 1 min	5	[110]

Orange juice	*E. coli, L. innocua *	241 MPa, 3 min	5	[111]

**Table 5:** Effect of high pressure homogenization on microorganisms in fruit juices.

Fruit juice(s)	Target microorganisms	Treatment parameters	Log reduction	References
Orange juice	*Escherichia coli *O58:H21 ATCC 10536 *E. coli *O157:H7 CCUG 44857	300 MPa	4	[112]

Orange juice	*Saccharomyces cerevisiae, Lactobacillus plantarum *	>250 MPa	>5	[113]

Apple juice	*Saccharomyces, Penicillium, Aureobasidium*, and *Aspergillus *	300 MPa	>5	[114]

Apricot juice	*S. cerevisiae *	100 MPa (4 times)	2.2	[115]

Carrot juice	*S. cerevisiae *	100 MPa (3 times)	3	[115]

Apricot juice	*Zygosaccharomyces bailli *	100 MPa (8 times)	2.5	[116]

Carrot juice	*Z. bailli *	100 MPa (8 times)	2.5	[116]

**Table 6:** Effect of pulsed electric field on microorganisms in fruit juices.

Fruit juice(s)	Target microorganisms	Treatment parameters	Log reduction	References
Apple juice	*Escherichia coli *8739, *E. coli *O157:H7	30 kV/cm, 172 *μ*s, <35°C	5	[117]

Cranberry juice	Total aerobic count, moulds, and yeasts	40 kV/cm, 150 *μ*s, <25°C	4	[118]

Apple juice	*E*. *coli* O157:H7	34 kV/cm, 166 *μ*s	4.5	[119]

Orange juice	*Listeria innocua *	30 kV/cm, 12 *μ*s, 54°C	6.0	[120]

Apple cider	*E. coli *O157:H7	90 kV/cm, 20 *μ*s, 42°C	5.9	[121]

Orange juice	*Salmonella typhimurium *	90 kV/cm, 100 *μ*s, 55°C	5.9	[122]

Apple juice	*E. coli *	34 kV/cm, 7.68 *μ*s, 55°C	6.2	[123]

Grape juice	*E. coli *	34 kV/cm, 7.68 *μ*s, 55°C	6.4	[123]

Orange juice	*Lactobacillus brevis *	35 kV/cm, 2.5–5.0 *μ*s	5.8	[124]

Orange juice-milk mixture	*L. plantarum *	35 kV/cm, 2.5–5.0 *μ*s	2.5	[125]

Apple juice	*E. coli *	36 kV/cm, 800 pulse s per second	6	[77]

Cherry juice	*Penicillium expansum *	34 kV/cm, 163 *μ*s, 21°C	100% inactivation of spore germination	[126]

Orange juice	*Salmonella enteritidis, E. coli *	35 kV/cm, 4 *μ*s, 40°C	5	[17]

Strawberry juice	*E. coli *O157:H7	18.6 kV/cm, 150 *μ*s, 45°C	3.09	[79]
		18.6 kV/cm, 150 *μ*s, 50°C	4.08	
		18.6 kV/cm, 150 *μ*s, 55°C	4.71	

**Table 7:** Effect of ultraviolet on microorganisms in fruit juices.

Fruit juice	Target microorganisms	Treatment parameters mJ/cm^2^	Log reduction	References
Apple juice	*Escherichia coli *K12	14.5	3-4	[127]
Orange juice	Yeasts, moulds	12.3–120	3	[128]
Apple juice	*Saccharomyces cerevisiae*,* Listeria innocua*, and *E. coli *	5135	1.34 4.29 5.10	[129]
Apple juice	∗APC	229.5 J/L	3.5	[81]
Guava pineapple	Yeasts	1377 J/L	3.0	
	Moulds		4.48	
Orange juice 1	APC	167 J/L	1.32	
	Yeasts			
	Moulds			
Orange juice 2	APC	167 J/L	<1	
	Yeasts			
	Moulds			

APC: aerobic plate count.

**Table 8:** Effect of ultrasound on microorganisms in fruit juices.

Fruit juice(s)	Target microorganisms	Treatment parameters	Log reduction	References
Carrot juice	*Escherichia coli *K12	19.3 kHz, 700–800 W, 1 min, 60°C	2.5	[130]

Orange juice	Total mesophilic Aerobes	500 kHz, 240 W, 15 min, 60°C	3.4	[131]

Apple juice	*Alicyclobacillus* *acidoterrestris *	24 kHz, 300 W, 60 min	80%	[132]

Orange juice	Aerobic mesophilic count (AMC)	20 kHz, 500 W, 8 min, 10°C	1.38	[133]
	Yeast and mold counts (YMC)		0.56	

**Table 9:** Effect of natural antimicrobials on spoilage and pathogenic microorganisms.

Natural antimicrobial	Fruit juice	Target microorganisms	Log reduction	References
Lactoperoxidase	Apple and orange juice	*Escherichia coli, Shigella *sp.	>5	[64]
Lysozyme	Orange juice	*Salmonella typhimurium *	—	[122]
Chitosan	Apple juice	Yeasts and moulds	3	[134]
		Yeasts	3–5	[135]
Cinnamon powder	Apple juice	*Listeria monocytogenes Scott 45954 *	4–6	[136]
Essential oil				
Cinnamon oil	Apple, pear, melon juices	*L. innocua, S. enteritidis*, *E. coli *	5	[137]
	Melon, watermelon	*S. enteritidis *	3.1–3.9	[17]
		*E. coli *	1.4–1.9	
		*L. monocytogenes *	3.4–4.4	
	Apple juice	*S. hadar*, *E*. *coli* O157:H7	50%	[138]
Clove oil	Apple juice	*S. hadar*, *E*. *coli* O157:H7	50%	[138]
	Tomato juice	Native microbiota	3.9	[139]
Lemon oil	Apple juice	*S*. *hadar*, *E*. *coli* O157:H7	50%	[138]
Lemongrass oil	Apple, pear, melon juices	*L. innocua, S. enteritidis, E. coli *	5	[137]
	Apple juice	*S. hadar*, *E*. *coli* O157:H7	50%	[138]
Lime oil	Apple juice	*S. hadar*, *E*. *coli* O157:H7	50%	[138]
Oregano oil	Apple juice	*S. hadar*, *E*. *coli* O157:H7	50%	[138]
Carvacrol	Apple juice	*S. hadar*, *E*. *coli* O157:H7	50%	
Cinnamaldehyde	Apple juice	*S. hadar*, *E*. *coli* O157:H7	50%	[139]
Citral	Apple juice	*S*. *hadar*, *E*. *coli* O157:H7	50%	[138]
	Orange juice	*L. monocytogenes *	1.1–1.3	[140]
Eugenol	Apple juice	*S. hadar*, and *E*. *coli* O157:H7	50%	[138]

**Table 10:** Preservation of fruit juices by combination of different preservation methods.

Fruit juice	Combination of preservation methods	Target microorganisms	Log reduction	References
Orange juice	PEF with nisin	Native microflora	6	[141]

Orange juice	PEF with nisin or lysozyme	*Escherichia coli* O157:H7	>7	[122]

Strawberry juice	PEF with cinnamon bark oil or citric acid	*E. coli* O157:H7, *Salmonella enteritidis *	>5	[17]

Apple juice	PEF with cinnamon bark oil or citric acid	*E. coli* O157:H7, *S. enteritidis *	>5	[17]

Pear juice	PEF with cinnamon bark oil or citric acid	*E. coli* O157:H7, *S. enteritidis *	>5	[17]

Carrot juice	HPH with nisin	*L. innocua *	>5	[142]

Strawberry juice	PEF with sodium benzoate and potassium sorbate	*E. coli* O157:H7, *S. enteritidis *	5.11	[79]
